# Rapamycin Inhibits Oxidized Low Density Lipoprotein Uptake in Human Umbilical Vein Endothelial Cells via mTOR/NF-κB/LOX-1 Pathway

**DOI:** 10.1371/journal.pone.0146777

**Published:** 2016-01-11

**Authors:** Yan-De Zhou, Xue-Qin Cao, Zhi-Hua Liu, Yong-Jun Cao, Chun-Feng Liu, Yan-Lin Zhang, Ying Xie

**Affiliations:** 1 Department of Endocrinology, The Second Affiliated Hospital of Soochow University, Suzhou, Jiangsu, China; 2 Department of Neurology, The Second Affiliated Hospital of Soochow University, Suzhou, Jiangsu, China; 3 Institute of Neuroscience, Soochow University, Suzhou, Jiangsu, China; School of Medicine, University of Belgrade, SERBIA

## Abstract

**Background:**

Lectin-like oxidized low-density lipoprotein-1 (LOX-1) is the major receptor for oxidized low density lipoprotein (ox-LDL) uptake in human umbilical vein endothelial cells (HUVECs). Previously, we found that rapamycin inhibited ox-LDL accumulation in HUVECs, and this effect was related to its role in increasing the activity of autophagy-lysosome pathway. In this study, we determined whether rapamycin could also reduce ox-LDL uptake in HUVECs and investigated the underlying signaling mechanisms.

**Results:**

Flow cytometry and live cell imaging showed that rapamycin reduced Dil-ox-LDL accumulation in HUVECs. Furthermore, rapamycin reduced the ox-LDL-induced increase in LOX-1 mRNA and protein levels. Western blotting showed that rapamycin inhibited mechanistic target of rapamycin (mTOR), p70s6k and IκBα phosphorylation triggered by ox-LDL. Flow cytometry implied that mTOR, NF-κB knockdown and NF-κB inhibitors significantly reduced Dil-ox-LDL uptake. Moreover, immunofluorescent staining showed that rapamycin reduced the accumulation of p65 in the nucleus after ox-LDL treatment for 30 h. mTOR knockdown decreased LOX-1 protein production and IκBα phosphorylation induced by ox-LDL. NF-κB knockdown and NF-κB inhibitors reduced LOX-1 protein production, but did not inhibit mTOR phosphorylation stimulated by ox-LDL.

**Conclusions:**

These findings demonstrate that rapamycin reduce mTOR phosphorylation and subsequently inhibit NF-κB activation and suppresses LOX-1, resulting in a reduction in ox-LDL uptake in HUVECs.

## Introduction

Endothelial dysfunction is a very early step in atherosclerosis (AS), which is one of the most common pathological manifestations of vascular disease. The interaction between lipoproteins and endothelial cells plays a crucial role in the generation and development of AS [1]. Oxidized low-density lipoprotein (ox-LDL) is the oxidized product of low-density lipoprotein (LDL), and is a major risk factor for the pathogenesis of AS. Absorbed into the cells of vascular walls through lectin-like oxidized low-density lipoprotein-1 (LOX-1) and other scavenger receptors (SRs), ox-LDL can induce the formation of foam cells and exacerbate vascular inflammation [2–4].

The rapid, unregulated uptake of ox-LDL by SRs is crucial for the transformation of macrophages to foam cells in atherosclerotic lesions [5]. However, LOX-1, a kind of SRs, does not share any homology with other SRs. Ling et al demonstrated that 70% ox-LDL uptake in endothelial cells was regulated by LOX-1, and only 30% was dependent on other SRs [6]. LOX-1 was originally identified as the primary receptor for ox-LDL uptake in endothelial cells [7]. The cell-surface receptor LOX-1 participates in the binding, endocytosis, and proteolytic degradation of ox-LDL.

Mechanistic target of rapamycin (mTOR) is well known for inducing autophagy by inhibiting mTOR and also serves as an immunosuppressant in the treatment of cancer. Increasing evidences indicate that mTOR inhibition stabilizes atherosclerotic plaques and limits atherosclerotic progression *in vitro* [8, 9] and *in vivo* [10, 11], however, our understanding of the mechanism remains incomplete. We intend to explore that mechanism and have previously demonstrated that rapamycin can decrease ox-LDL aggregation in human umbilical vein endothelial cells (HUVECs) by inducing autophagy [12]. It is well known that the toxic effect of ox-LDL is associated with problems with its degradation following oxidation [13]. Ox-LDL aggregation in cells is influenced not only by problems with its degradation, but also associated with the increase in its endocytosis. Therefore, we sought to ascertain whether rapamycin can also reduce the endocytosis of ox-LDL in HUVECs and to investigate the underlying signaling mechanisms.

## Material and Methods

### Reagents and antibodies

Ox-LDL and 1, 1′-dioctadecyl-3, 3, 3′, 3′-tetramethyl-indocarbocyanine perchlorate (Dil)-labeled ox-LDL (Dil-ox-LDL) were purchased from Yiyuan Biotechnologies (Guangzhou, China). Chemicals and the primary antibodies against SQSTM1 (p62), glyceraldehyde 3-phosphate dehydrogenase (GAPDH) and β-tubulin were obtained from Sigma-Aldrich (Saint. Louis, MO, USA). Other primary antibodies were purchased from Cell Signaling Technology (Danvers, MA, USA). Reagents for cell culture were obtained from Gibco (Grand Island, USA).

### Cell culture

HUVECs were purchased from Shanghai Gene Chemical Co., Ltd (Shanghai, China). They were cultured in six-well culture plates and propagated in high-glucose Dulbecco’s Modified Eagle’s Medium (DMEM) supplemented with 10% fetal bovine serum (FBS). The cells were maintained in a gas mixture of 5% CO_2_ and 95% air at 37°C. HUVECs cultured in passage numbers 3–10 were used in the experiments and were subcultured every 48 h by using 0.25% trypsin.

### MTT assay

Cell viability was measured using a colorimetric assay with 3-(4,5-dimethylthiazol-2-yl)-2,5-diphenyltetrazolium bromide (MTT). The dehydrogenase enzymes of the intact mitochondria of living cells transform MTT into insoluble formazan. The formazan was dissolved in dimethylsulfoxide(DMSO), and the absorbance value was determined with a microplate reader(Tecan) at 490 nm. The absorbance value of each group was normalized to the value of the control cells.

### Flow cytometry

Cells were harvested at 6 h after being co-cultured with Dil-ox-LDL. Briefly, the cells were washed twice with 1×phosphate-buffered saline (PBS). Furthermore, 1×10^5^ HUVECs were resuspended in a final volume of 300 μL 1× PBS. The rate of Dil-ox-LDL uptake in HUVECs was measured using a flow cytometer (FC500; Beckman Coulter, Miami, FL, USA) with excitation-emission wavelengths of 549 nm: 565 nm.

### Live cell imaging

HUVECs were seeded in special glass culture dishes for real-time cell imaging. Image acquisition was started the moment Dil-ox-LDL was added to the culture dishes. Experiments were conducted using a live-cell imaging system (cell^R; Olympus, Tokyo, Japan). Time-lapse images were acquired every 5 min for 12 h using an oil-immersed 60× objective lens.

### Quantitative polymerase chain reaction (q-PCR)

Total RNA was extracted with TRIzol (Invitrogen, Carlsbad, CA, USA). Each sample was reversely transcribed into complementary DNA (cDNA) using a cDNA Synthesis kit (Fermentas, Vilnius, Lithuania) according to the manufacturer’s instructions. Q-PCR were performed on the ABI 7500 system (Applied Biosystems, Foster City, CA, USA) using an SYBR^®^ green PCR Master Mix (Invitrogen, Carlsbad, CA, USA), and the following primers (GenScript, Nanjing, China): human LOX-1 (forward, 5′-TTACTCTCCATGGTGGTGCC-3′; reverse, 5′-AGCTTCTTCTGCTTGTTGCC-3′) and 18S (forward: 5′-TCAACACGGGAAACCTCAC-3′; reverse: 5′-CGCTCCACCAACTAAGAAC-3′). The cycle time values were normalized to18S of the same sample. The mRNA levels were then reported relative to the control levels.

### Western blotting analysis

Cell lysates were prepared using lysis buffer (150 mM NaCl, 25 mM Tris, 5 mM EDTA and 1% Nonidet P-40; pH 7.5) with protease inhibitor cocktail tablets (Roche Diagnostics, Penzberg, Germany) and phosphorylated protease inhibitor NaF. HUVEC lysates were resolved by sodium dodecyl sulfate-polyacrylamide gel electrophoresis and transferred to polyvinylidene difluoride (PVDF) membranes. After blocking in 5% bovine serum albumin (BSA), the proteins were immunoblotted with primary antibodies, followed by horseradish peroxidase-linked secondary antibodies. Detection was carried out using ECL reagent, and the relative intensities of protein bands were analyzed using Image J software.

### Transient transfection

The small-interfering RNAs (siRNA) mTOR (forward:5′-GCCGCAUUGUCUCUAUCAATT-3′; reverse: 5′-UUGAUAGAGACAAUGCGGCTT-3′), NF-κB (forward:5′-CCUGGAACUACUAAAUCUATT-3′; reverse:5′-UAGAUUUAGUAGUAGUUCCAGGTT-3′), LOX-1 (forward:5′-GGAUGAGUUUAGCCACUAUTT-3′; reverse:5′- AUAGUGGCUAAACUCAUCCTT-3′) and the scrambled siRNA duplexes were synthesized by GenePharma(Shanghai, China). siRNA duplexes and mTOR/NF-κB plasmids were transfected using HiPerFect Transfection Reagent and Effectene Transfection Reagent, respectively (Qiagen, Stanford, VA, USA). Transfection efficiency was determined by western blotting after transfection for 24 h.

### Immunofluorescent staining

Cells were fixed with 4% paraformaldehyde for 10 min and permeabilized with 0.1%Triton X-100 in PBS for a further 8 min, followed by blocking with 5% BSA/PBS for 1 h. Afterward, the cells were incubated with rabbit anti-p65 (1:100 dilution) at 4°C overnight, then incubated with fluorescein isothiocyanate-conjugated goat anti-rabbit IgG (Invitrogen) in the dark for 1 h. Coverslips were then mounted using a mounting medium with 4',6-diamidino-2-phenylindole dihydrochloride (Vector Laboratories, Burlingame, CA, USA). Effects were observed and photographed using an Axio Scope A1 microscope (Zeiss, Göttingen, Germany).

### Statistical analysis

Data analyses were carried out using SPSS v17.0 (SPSS, Chicago, IL, USA) and GraphPad Prism v5.0. Results are shown as mean ± SEM. The significance of differences between two groups was determined using the Student’s t-test, and differences among groups were determined by analysis of variance (ANOVA). P < 0.05 was considered significant.

## Results

### Rapamycin reduces Dil-ox-LDL accumulation in HUVECs

**Fig 1:**
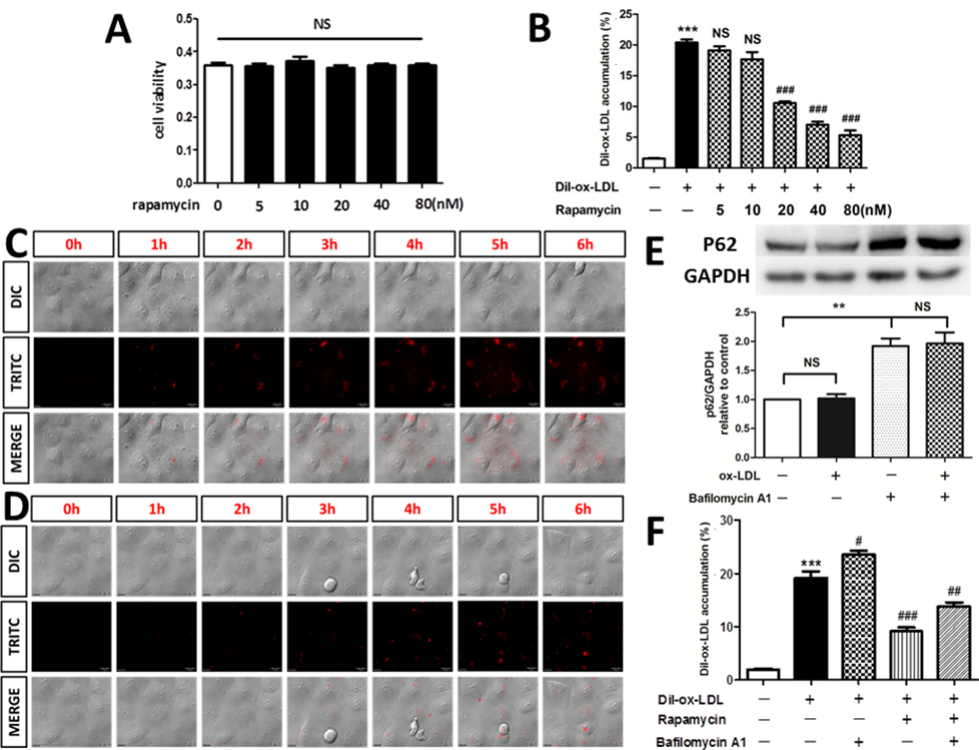
Effect of rapamycin on Dil-ox-LDL uptake in HUVECs. (A) MTT assay for cell viability after treatment with rapamycin by concentration course. NS = not significant. Data are expressed as mean ± SEM, n = 3. (B) Flow cytometry showed that pretreatment with at least 20 nM rapamycin for 1h significantly reduced Dil-ox-LDL accumulation in HUVECs. ***P < 0.001 versus control; ###P < 0.001 versus Dil-ox-LDL group; NS = not significant compared with the Dil-ox-LDL group. Data are expressed as mean ± SEM, n = 3. (C) Continuous images obtained from live cell imaging after 30 μg/mL Dil-ox-LDL treatment for 6 h. (D) Continuous images obtained from live cell imaging after 30 μg/mL Dil-ox-LDL treatment for 6 h following pretreatment with 20 nM rapamycin for 1 h. The experiment was repeated independently three times. (E)Western blot indicated that p62 was increased by bafilomycin A1, and was not reduced by simultaneous treatment with ox-LDL. NS = not significant. **P < 0.01 versus bafilomycin A1 group. Data are expressed as mean ± SEM, n = 3. (F)Flow cytometry indicated that after pretreatment with bafilomycin A1, rapamycin could still inhibit Dil-ox-LDL uptake in HUVECs. ***P < 0.001 versus control; #P < 0.05 versus Dil-ox-LDL group; ###P < 0.001 versus Dil-ox-LDL group; ##P < 0.01 versus Dil-ox-LDL group. Data are expressed as mean ± SEM, n = 3.

The MTT assay showed that 5~80 nM rapamycin treatment for 7 h did not adversely affect cell viability (Fig 1A). The results of flow cytometry indicated that Dil-ox-LDL accumulation reduced significantly after pretreatment with at least 20 nM rapamycin for 1 h followed by 30 μg/mL Dil-ox-LDL for 6 h (Fig 1B). A similar result for Dil-ox-LDL uptake was observed by live cell imaging. Continuous images demonstrated that without rapamycin pretreatment, 30 μg/mL Dil-ox-LDL fluorescence intensity increased as time passed (Fig 1C). However, after pretreatment with 20 nM rapamycin for 1 h followed by treatment with 30 μg/mL Dil-ox-LDL (Fig 1D), the red fluorescent of Dil-ox-LDL was not as obvious as in Fig 1C. To exclude the effect of degradation in intracellular ox-LDL accumulation, we used bafilomycin A1 to inhibit the completion of autophagy. The result of western blot showed that the protein level of the selective autophagic target p62 was increased by bafilomycin A1, and was not reduced by simultaneous treatment with ox-LDL, which suggested that bafilomycin A1 was able to completely block autophagy (Fig 1E). Moreover, the result of flow cytometry indicated that after pretreatment with 50 nM bafilomycin A1 for 1 h, rapamycin could still inhibit Dil-ox-LDL accumulation in HUVECs, which showed that besides the effect of degradation, other effects mediated by rapamycin also participated in the accumulation of Dil-ox-LDL in HUVECs (Fig 1F).

### Rapamycin suppresses expression of LOX-1 mRNA and protein in HUVECs

**Fig 2:**
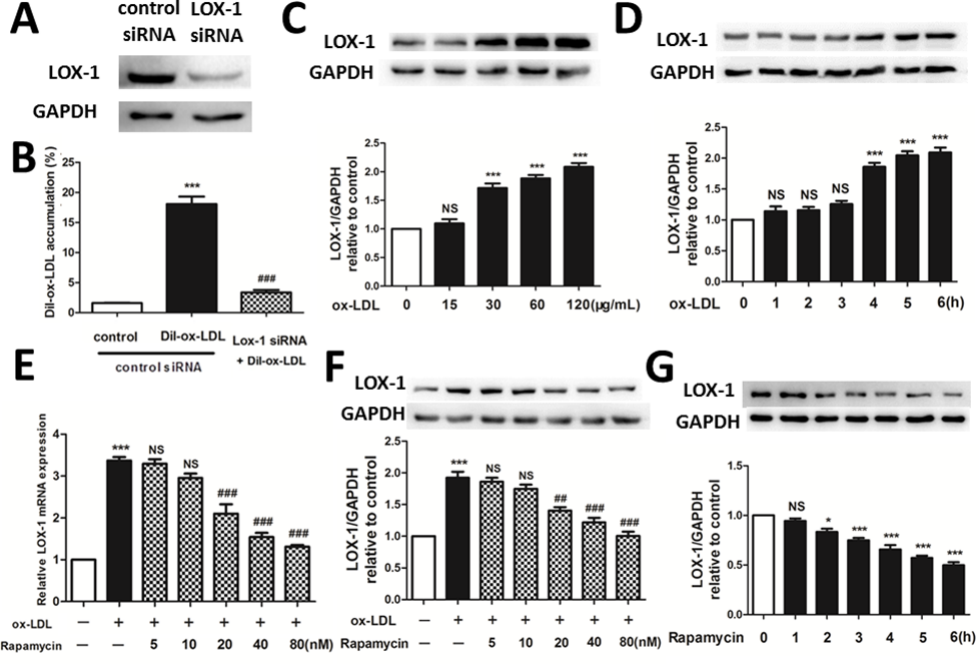
Effect of rapamycin on LOX-1 expression in HUVECs. (A, B) Knocking down LOX-1 significantly reduced the rate of Dil-ox-LDL uptake in HUVECs. ***P < 0.001 versus control; ###P < 0.001 versus ox-LDL group. Data are expressed as mean ± SEM, n = 3. (C, D) The expression of LOX-1 protein assessed by western blotting after treatment of HUVECs with ox-LDL by time and concentration course. (E) The q-PCR showed that at least 20 nM rapamycin reduced the increase in LOX-1 mRNA expression induced by ox-LDL. ***P < 0.001 versus control; ###P < 0.001 versus Dil-ox-LDL group; NS = not significant compared with the Dil-ox-LDL group. Data are expressed as mean ± SEM, n = 3. (F) Western blotting indicated that rapamycin dose-dependently reduced the increase in production of LOX-1 protein triggered by ox-LDL. GAPDH was used as the loading control. ***P < 0.001 versus control; #P < 0.05 versus ox-LDL group; ###P < 0.001 versus ox-LDL group; NS = not significant compared with the ox-LDL group. Data are expressed as mean ± SEM, n = 3. (G) Western blotting indicated that 20 nM rapamycin time-dependently reduced the production of LOX-1 protein. *P < 0.05 versus control; ***P < 0.001 versus control; NS = not significant compared with the control group. Data are expressed as mean ± SEM, n = 3.

Knocking down LOX-1 significantly reduced the rate of Dil-ox-LDL uptake in HUVECs, confirming that LOX-1 played a vital role in ox-LDL uptake in HUVECs (Fig 2A and 2B). To keep pace with the concentration and stimulation time of Dil-ox-LDL used in flow cytometry (20–40 μg/mL incubated for 3–6 h recommended by the manufacturer), incubation with 30 μg/mL ox-LDL for 6 h was also carried out in the present study. It was confirmed that this culture condition also significantly increased the expression of LOX-1 protein by concentration and time course of ox-LDL (Fig 2C and 2D). Studies have shown that SRs are expressed in very small amounts on vascular endothelial cells [14]. However, endothelial cells express large amounts of LOX-1 [15], suggesting that LOX-1 is the major receptor expressed on endothelial cells for ox-LDL uptake. The q-PCR showed that pretreatment with a minimum dose of 20 nM rapamycin for 1 h significantly reduced production of LOX-1 mRNA in ox-LDL-stimulated HUVECs (Fig 2E). Consistent with the PCR data, western blotting confirmed that treatment with 20 nM rapamycin for 1 h significantly suppressed the expression of LOX-1 protein induced by ox-LDL treatment (Fig 2F). Moreover, treatment with 20 nM rapamycin for at least 2 h individually inhibited the expression of LOX-1 protein (Fig 2G).

### Rapamycin reduces ox-LDL uptake by inhibiting mTOR phosphorylation

**Fig 3:**
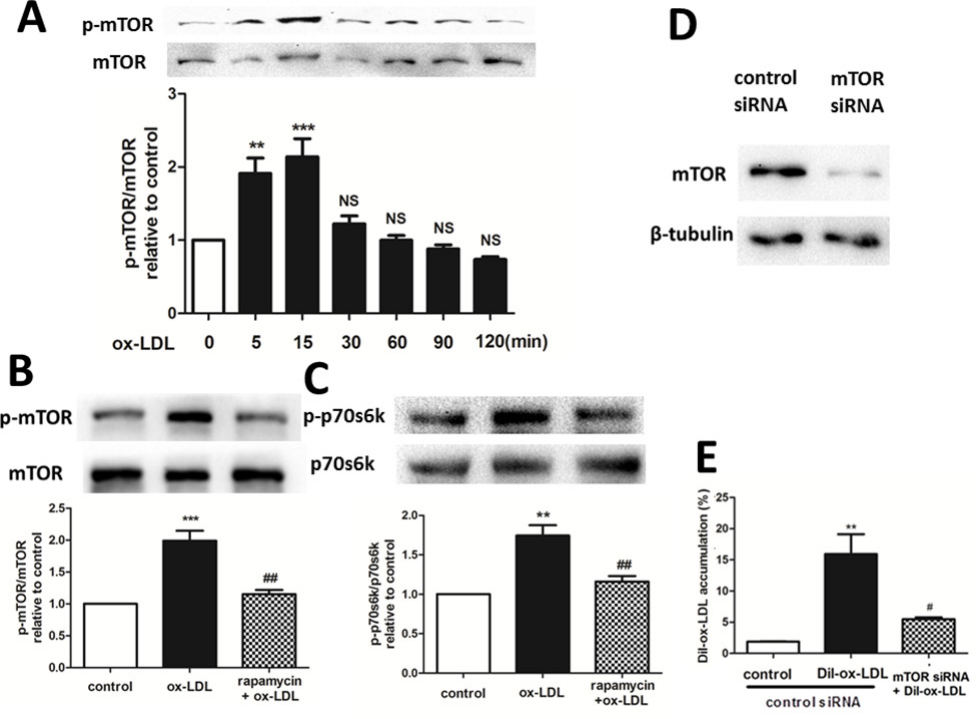
Effect of the mTOR/p70s6k signaling pathway on ox-LDL uptake in HUVECs. (A, B) Phosphorylation and degradation of mTOR analyzed by western blotting. **P < 0.01 versus control; ***P < 0.001 versus control; NS = not significant compared with the control group. Data are expressed as mean ± SEM, n = 3. (C) p70s6k phosphorylation analyzed by western blotting. **P < 0.01 versus control; ## P < 0.01 versus ox-LDL group. Data are expressed as mean ± SEM, n = 3. (D-E) Flow cytometry showed that knockdown of mTOR significantly reduced Dil-ox-LDL uptake in HUVECs. **P < 0.01 versus control; #P < 0.05 versus Dil-ox-LDL group. Data are expressed as mean ± SEM, n = 3.

Ox-LDL induced an increase in mTOR phosphorylation in a time-dependent manner, and the maximal effect was achieved at 15 min after 30 μg/mL ox-LDL treatment (Fig 3A). mTOR is the target for combining with rapamycin. Rapamycin serves as an autophagy activator by inhibiting mTOR, as corroborated by the result of our western blotting analysis of HUVECs (Fig 3B). Expression of a downstream protein of mTOR, the 70-kDa ribosomal protein S6 kinase (P70s6k), also increased 15 min after 30 μg/mL ox-LDL treatment, and was inhibited by pretreatment with 20 nM rapamycin for 1 h, which also suggested that the activity of mTOR protein was inhibited by rapamycin (Fig 3C). Moreover, mTOR knockdown significantly reduced the rate of Dil-ox-LDL uptake in HUVECs, implying that rapamycin reduces ox-LDL uptake by inhibiting mTOR phosphorylation (Fig 3D and 3E).

### Rapamycin reduces ox-LDL uptake by inhibiting NF-κB activation

**Fig 4:**
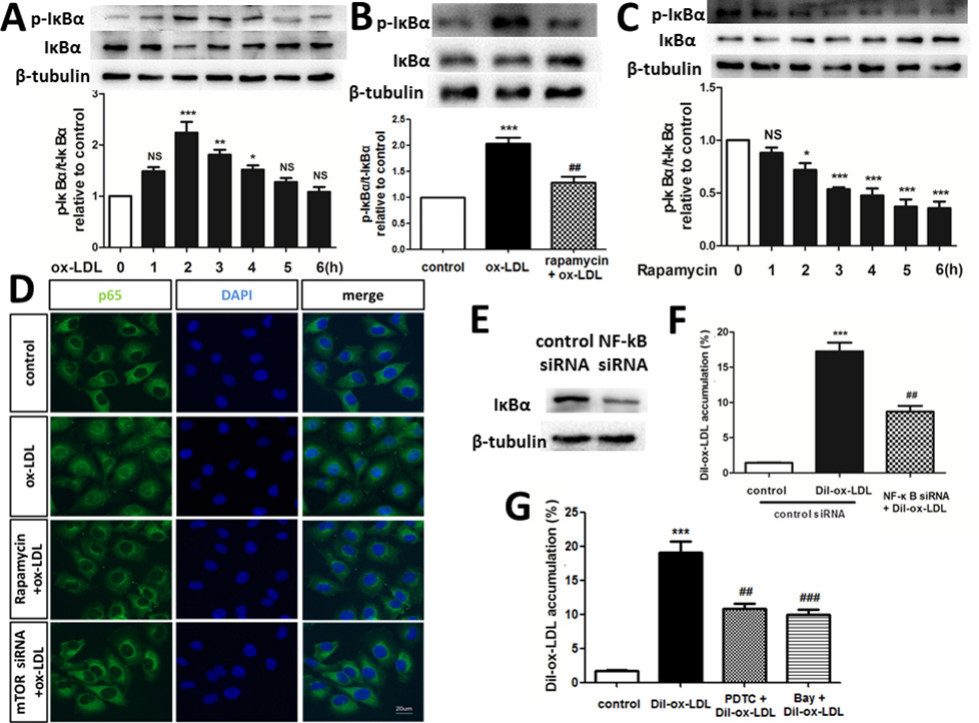
Effect of the P65/NF-κB signaling pathway on ox-LDL uptake in HUVECs. (A, B) Phosphorylation and degradation of IκBα analyzed by western blotting. *P < 0.05 versus control; **P < 0.01 versus control; ***P < 0.001 versus control; ##P < 0.01 versus ox-LDL group; NS = not significant compared with the control group. Data are expressed as mean ± SEM, n = 3–4. (C) Western blotting indicated that rapamycin time-dependently inhibited the phosphorylation of IκBα. *P < 0.05 versus control; ***P < 0.001 versus control; NS = not significant compared with the control group. Data are expressed as mean ± SEM, n = 3. (D) Translocation of NF-κB p65 was observed after ox-LDL treatment for 30 h under a fluorescence microscope. The experiment was repeated independently three times. (E-G) Flow cytometry showed that inhibition of NF-κB significantly reduced Dil-ox-LDL uptake in HUVECs. ***P < 0.001 versus control; ##P < 0.01 versus Dil-ox-LDL group; ###P < 0.001 versus Dil-ox-LDL group. Data are expressed as mean ± SEM, n = 3.

Rafiee et al demonstrated that rapamycin inhibited NF-κB activation triggered by radiation in human intestinal microvascular endothelial cells [16]. In the present study, ox-LDL increased IκBα phosphorylation in a time-dependent manner, for which a maximal effect was achieved 2 h after 30 μg/mL ox-LDL treatment (Fig 4A). This effect was abolished by pretreatment with 20 nM rapamycin for 1 h (Fig 4B). Moreover, western blotting showed that treatment with 20 nM rapamycin for at least 2 h inhibited NF-κB expression in HUVECs (Fig 4C). Immunofluorescence staining (Fig 4D) revealed that p65 was located mainly in the cytoplasm in vehicle-treated HUVECs. A remarkable accumulation of p65 in the nucleus was observed after treatment with 30 μg/mL ox-LDL for 30 h suggesting that ox-LDL induces p65 redistribution from the cytoplasm to the nucleus, whereas this effect was alleviated in the presence of rapamycin and mTOR siRNA. Moreover, NF-κB knockdown and NF-κB inhibitors, pyrrolidine dithiocarbamate (PDTC) and Bay, significantly reduced the rate of Dil-ox-LDL uptake in HUVECs, implying that rapamycin reduces ox-LDL accumulation by inhibiting NF-κB activation (Fig 4E–4G).

### mTOR siRNA inhibits NF-κB activation, resulting in the suppression of LOX-1 expression

**Fig 5:**
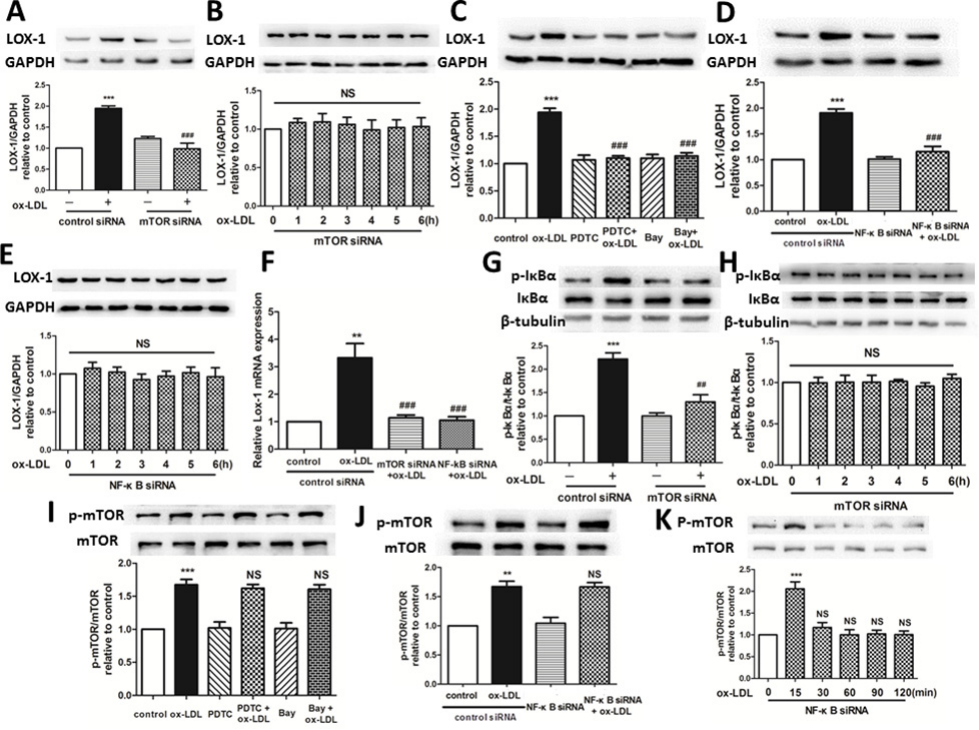
Upstream and downstream relationship between mTOR, NF-κB, LOX-1 and ox-LDL uptake in HUVECs. (A, B) Western blotting showed that inhibition of mTOR significantly reduced the increase in the expression of LOX-1 protein expression induced by ox-LDL. ***P < 0.001 versus control; ###P < 0.001 versus ox-LDL; NS = not significant. Data are expressed as mean ± SEM, n = 3. (C-E) Western blotting showed that inhibition of NF-κB significantly reduced the increase in the expression of LOX-1 protein expression induced by ox-LDL. ***P < 0.001 versus control; ###P < 0.001 versus ox-LDL; NS = not significant. Data are expressed as mean ± SEM, n = 3. (F) q-PCR indicated that mTOR and NF-κB knockdown reduced the upregulated expression of LOX-1 mRNA induced by ox-LDL. **P < 0.01 versus control; ###P < 0.001 versus ox-LDL. Data are expressed as mean ± SEM, n = 3. (G, H) IκBα phosphorylation and (I-K) mTOR phosphorylation were analyzed by western blotting, which showed that mTOR deficiency significantly reduced the IκBα phosphorylation triggered by ox-LDL, whereas inhibition of NF-κB did not reduce mTOR phosphorylation induced by ox-LDL. **P < 0.01 versus control; ***P < 0.001 versus control; ##P < 0.01 versus ox-LDL group; NS = not significant. Data are expressed as mean ± SEM, n = 3.

To ascertain the specific signaling mechanism by which rapamycin reduces Dil-ox-LDL uptake in HUVECs, upstream and downstream relationships were investigated. mTOR siRNA reduced the expression of LOX-1 protein induced by 30 μg/mL ox-LDL treatment for 6 h (Fig 5A). Moreover, knocking down mTOR also reduced the expression of LOX-1 protein which was expected to have been increased by 30 μg/mL ox-LDL treatment for 4–6 h (Fig 5B). NF-κB inhibitors (PDTC and Bay) and NF-κB siRNA also reduced the expression of LOX-1 protein induced by 30 μg/mL ox-LDL treatment for 6 h (Fig 5C and 5D). Knocking down NF-κB also reduced the expression of LOX-1 protein, which was expected to have been increased by 30 μg/mL ox-LDL treatment for 4–6 h (Fig 5E). Both mTOR and NF-κB knockdown reduced the upregulation of LOX-1 mRNA expression induced by 30 μg/mL ox-LDL treatment for 6 h (Fig 5F). Moreover, mTOR knockdown inhibited the IκBα phosphorylation induced by 30 μg/mL ox-LDL treatment for 2 h (Fig 5G). Knocking down mTOR also inhibited IκBα phosphorylation, which was expected to have been induced by 30 μg/mL ox-LDL treatment for 2–4 h (Fig 5H). However, NF-κB inhibitors (PDTC and Bay) and NF-κB siRNA did not inhibit mTOR phosphorylation 15 min after 30 μg/mL ox-LDL treatment (Fig 5I and 5J). Knocking down NF-κB did not inhibit mTOR phosphorylation induced by ox-LDL treatment at 15 min (Fig 5K). These results imply that mTOR knockdown leads to the inhibition of downstream activation of NF-κB protein, followed by the suppression of LOX-1 protein expression.

## Discussion

Besides macrophages, lipoprotein particles accumulating below vascular endothelial cells are taken up and degraded by those cells. Endothelial cells also play an essential role in preventing lipoprotein particles from being absorbed into the intima [17]. Reducing the accumulation of ox-LDL in HUVECs is important for preventing the development of AS.

Rapamycin is an inhibitor of mTOR phosphorylation. In a previous study using flow cytometry and live cell imaging, we found that rapamycin could also inhibit ox-LDL accumulation in HUVECs, and this effect was related to its role in increasing autophagic degradation of ox-LDL [12]. However, in this study, we found that after using bafilomycin A1 to inhibit the completion of autophagy, rapamycin could still inhibit Dil-ox-LDL accumulation in HUVECs, which showed that besides the effect of degradation, other mechanism might involve in the rapamycin-mediated decrease of Dil-ox-LDL accumulation in HUVECs. As we all know, the accumulation of ox-LDL in HUVECs is not only related to the degradation-based disorders of ox-LDL, but also to the increase in ox-LDL uptake. Therefore, it’s necessary to assess the effect of uptake on ox-LDL accumulation in HUVECs.

LOX-1 is the major receptor for ox-LDL uptake in endothelial cells. It has also been shown that multiple disease states, such as AS, hypertension, hyperlipidemia, diabetes mellitus, and ischemia reperfusion injury, involve upregulated expression of LOX-1 [18]. Under hyperlipidemic conditions, augmented uptake of ox-LDL and inflammatory cytokines as well as accelerated generation of atheroma-like lesions have been observed in LOX-1 overexpressing mice crossed with apolipoprotein E-null (LOXtg/apoEKO) mice [19]. In addition, a reduction in the number of AS lesions was found in LOX-1^-/-^/LDL-R^-/-^ mice compared with LDLR knockout mice. Moreover, ApoE knockdown mice displayed a significant increase in plaque coverage after LOX-1 overexpression, compared with that observed in the controls [20]. Our work showed that the expression of LOX-1 mRNA and protein in HUVECs increased after ox-LDL treatment for 6 h, and this effect was inhibited by rapamycin pretreatment. These results imply that the effect of rapamycin on inhibiting ox-LDL uptake is related to its role in decreasing LOX-1 production.

As a highly conserved ser/thr kinase, mTOR plays a crucial role in cell growth, proliferation, and survival [21]. Studies have identified that mTOR may be involved in AS. Animal models have indicated that mTOR inhibitors, such as sirolimus, attenuate inflammation, enhance the stability of atherosclerotic plaques [22] and influence the atherosclerotic process by affecting the accumulation of monocytes into early carotid lesions [23]. Moreover, another study has also demonstrated that sirolimus attenuates lipid accumulation and reduces the expression of inﬂammatory cytokines in vascular smooth muscle cells [24]. Coronary stents coated with sirolimus are widely used in revascularization procedures based on the evidence that sirolimus -eluting stents reduce neointimal proliferation, binary restenosis and the requirement for repeat revascularization compared with standard stents [25]. In our experiment, we have proved that ox-LDL treatment upregulates the level of mTOR phosphorylation in HUVECs, and that inhibiting mTOR phosphorylation by using mTOR siRNAs or rapamycin leads to reduced uptake of ox-LDL. These results imply that rapamycin may inhibit ox-LDL uptake in HUVECs by decreasing the level of mTOR phosphorylation.

Ox-LDL affects numerous signal transduction pathways that are dependent mainly on specific binding to the LOX-1 receptor. Of note, NF-κB activation induced by ox-LDL is crucial in the expression of vasoconstrictive molecules such as ET-1, and adhesion molecules including E- and P-selectins, vascular cell adhesion molecule-1 (VCAM-1), intercellular adhesion molecule-1 (ICAM-1), and monocyte chemotactic protein-1 (MCP-1) in endothelial cells [26–28]. One NF-κB binding site identified in the rat LOX-1 gene promoter region between nt-1494 and nt-1599 [29], suggested that NF-κB activation could induce the transcription of LOX-1 genes. Consistent with that study, our results showed that NF-κB activation inhibited by rapamycin resulted in a reduction in LOX-1 protein expression induced by ox-LDL. Ox-LDL binding to LOX-1 can increase the level of NADPH oxidase and reactive oxygen species (ROS), resulting in activation of redox-sensitive NF-κB. NF-κB activation subsequently induces LOX-1 expression, which in turn mediates more ox-LDL uptake, thereby constituting a “vicious circle” [15, 30].

The association between the mTOR signaling pathway and NF-κB activation has been investigated in many studies, but the results seem to be inconsistent and the underlying association may be more complicated. IκB kinases (IKKs) containing α, β, and γ subunits are the major protein kinases activating NF-κB in most signal transduction cascades. Among the three subunits, IKKα and β provide the catalytic activity of IKKs [31–33]. Dhingra et al. have determined that IKKβ overexpression results in mTOR activation, and conversely mTOR overexpression leads to NF-κB activation in ventricular myocytes [34]. Nevertheless, the authors of a different study have claimed that reduction of IKKβ inhibits the expression of mTOR and NF-κB [35]. With respect to the effect of regulation of the mTOR signaling pathway on NF-κB activation, the authors of some studies have verified that NF-κB activation is upregulated by mTOR [36–38], whereas other researchers consider the effects of mTOR to be due to down-regulation of NF-κB expression [39–41]. Therefore, we may conclude that the mTOR regulation of NF-κB differs in various environments, and is influenced by disparate conditions. In the present study, mTOR deficiency inhibited NF-κB activation, which was followed by reduced expression of the downstream protein LOX-1, suggesting that mTOR is upstream of NF-κB and that up-regulated NF-κB activation is induced by ox-LDL.

For the first time, this study confirmed the effect of rapamycin on ox-LDL uptake in HUVECs and linked mTOR, NF-κB and LOX-1 with this effect. Our future work will place particular emphasis on the specific mechanism underlying the mTOR/NF-κB/LOX-1 signaling pathway. The routine application of rapamycin as an immunosuppressant in AS patients is a distant goal, although many studies have proved its protective effect against AS. The role of mTOR in the progression of AS requires more attention, and the use of drugs that focus on suppressing mTOR may be a key breakthrough in the treatment of AS-related diseases.
